# Hydrogel increases diclofenac skin permeation and absorption

**DOI:** 10.1002/bdd.2194

**Published:** 2019-07-18

**Authors:** Eleonore Haltner‐Ukomadu, Manuel Sacha, Andrea Richter, Khaled Hussein

**Affiliations:** ^1^ Across Barriers GmbH Science Park 1, 66123 Saarbrücken Germany; ^2^ WITec GmbH Lise‐Meitner‐Strasse 6 D‐89081 Ulm Germany; ^3^ ratiopharm GmbH Graf‐Arco‐Strasse 3 89079 Ulm Germany

**Keywords:** diclofenac, emulsion gel, hydrogel, topical formulation, transdermal

## Abstract

**Purpose:**

Topical nonsteroidal anti‐inflammatory drug formulations are used commonly to treat musculoskeletal pain and inflammation. Drug properties and formulation composition are the primary determinants of the transdermal drug delivery rate. The *ex vivo* transdermal flux through human skin of three topical diclofenac formulations was compared.

**Methods:**

The formulations tested were hydrogel 1% diclofenac sodium and two emulsion gels (1.16%/2.32% diclofenac diethylamine, equivalent to 1%/2% diclofenac sodium). Human abdominal skin obtained during unrelated surgical procedures was stored at −20 °C until use. Skin specimens were thawed, prepared and placed in Franz diffusion cells (stratum corneum facing donor cell). The test formulation (~200 mg) was applied to the donor cell skin surface, and the receptor compartment was periodically sampled over 48 hours. The drug concentration in the receptor medium was determined by a validated HPLC method. Raman spectral imaging was performed to visualize the location and distribution of diclofenac.

**Results:**

After 5 hours, the cumulative amount of hydrogel diclofenac transiting the skin was about 10 times that of the emulsion gel 1.16% (*P*=0.0004) and about twice that of the emulsion gel 2.32% (*P*=0.022). Similar results were seen after 9 hours. Raman spectroscopy showed that the hydrogel formulation was a homogeneous mixture of its various components, including diclofenac. The emulsion gels were non‐homogeneous, with diclofenac in close proximity to the lipophilic (paraffin) phase.

**Conclusions:**

The transdermal transit of diclofenac from the hydrogel demonstrated a faster onset and a greater absorption rate than either emulsion gel formulation, suggesting that the hydrogel formulation may have a faster onset of action in underlying tissues vs. the emulsion gel products.

## 1 INTRODUCTION

Nonsteroidal anti‐inflammatory drugs (NSAIDs) are used commonly to treat pain and inflammation associated with acute and/or chronic musculoskeletal injuries and disorders (Bruyère et al., 2016; Smith, et al., 2016). Diclofenac (see Gan, 2010, for review) is the most commonly prescribed NSAID. In a survey of 15 low‐, middle‐ and high‐income countries, for example, diclofenac had a median market share of 27.8% in those countries, followed by ibuprofen (11.0%) and naproxen (9.4%) (McGettigan & Henry, 2013). Similarly, diclofenac is used widely in the United States, where more than 10.8 million prescriptions for diclofenac products were written in 2012 (Iroko Pharmaceuticals, 2014). Diclofenac is available in several types of dosing forms, including oral and injectable forms for systemic dosing and topical products for local treatment of underlying tissues (Altman, et at., 2015).

Topically applied NSAIDs can produce clinically effective drug concentrations at the site of action in the underlying tissue while reducing systemic exposure (Brunner et al., 2005; Miyatake, et al., 2009), which reduces the risk of systemic side effects and could improve patient compliance. The challenge in topical transdermal drug delivery is to create a formulation that allows the drug to permeate quickly and efficiently through the stratum corneum, the outermost layer of skin that provides the barrier function of the organ (Haftek, et at., 1998). The stratum corneum consists of 10–20 layers of cornified cells embedded in a hydrophobic lipid–protein matrix (Haftek, 2015). The primary pathway for absorption of topically applied drugs through the stratum corneum is thought to be this intercellular matrix (Vitorino, et al., 2015). Factors that influence the transdermal permeation of topically applied NSAID formulations include chemical structure and properties of the drug (e.g. molecular weight, hydrophilic vs. hydrophobic properties, free‐acid or free‐base vs. salt form of the molecule), and the composition of the formulation, particularly the inclusion of excipients that enhance dermal penetration (Brunner et al., 2011; Escribano, et al., 2003; Folzer, et al., 2014; Lane, 2013; Marwah, et al., 2016; Nivsarkar, et al., 2015; Vitorino et al., 2015).

The onset of a beneficial effect of an NSAID upon topical application is influenced by the time lag before the active drug is absorbed and the rate of flux of the drug through the skin. Here the results are presented of *ex vivo* testing of skin permeation using a well‐established method (Raney, et al., 2015) to compare three formulations of diclofenac and demonstrate that the hydrogel‐based formulation delivers diclofenac more quickly and at a higher rate than either of the emulsion gel formulations. In addition, Raman spectroscopy suggested differences in the location of diclofenac within the lipophilic and hydrophilic phases of each of the formulations that may contribute to the differences in transdermal drug transport.

## 2 MATERIAL AND METHODS

All experimental methods were performed using Good Laboratory Practice and all instruments used in the analyses described below conformed to this.

### 2.1 Diclofenac for topical administration formulations

Three different commercially available formulations of topical diclofenac were tested: hydrogel 1% (Olfen® Gel 1% [10 mg/g], Mepha, Aesch BL, Switzerland; Batch S09905), emulsion gel 1% (Voltaren Schmerzgel® 1.16%, Novartis Consumer Health GmbH, München, Germany; Batch WA865) and emulsion gel 2% (Voltaren Schmerzgel forte® 2.32%, Novartis Consumer Health GmbH, München, Germany; Batch K24512) (Novartis, 2013a, 2013b). The composition of each formulation is presented in Table 1. The active ingredient in the hydrogel 1% formulation is diclofenac sodium (10 mg/g), whereas the emulsion gel formulations use diclofenac diethylamine 1.16% or 2.32% (equivalent to 10 or 20 mg/g diclofenac sodium, respectively). The USP diclofenac sodium was purchased from LGC Ltd (Teddington, UK) and was used as the standard for all analytic testing.

**Table 1:** Composition of test formulations of transdermal diclofenac

	Hydrogel 1%	Emulsion gel 1%*	Emulsion gel 2%†
Diclofenac sodium	x		
Diclofenac diethylamine		x	x
Diisopropyl adipate	x		
Lactic acid	x		
Isopropyl alcohol	x	x	x
Sodium metabisulfite	x		
Hydroxypropyl cellulose	x		
Hydroxyethyl cellulose	x		
Purified water	x	x	x
Propylene glycol		x	x
Cocoyl caprylocaprate		x	x
Paraffin		x	x
Macrogol cetostearyl ether		x	x
Carbomer 974 P		x	x
Diethylamine		x	x
Perfume cream		x	
Oleyl alcohol			x
Eucalyptus‐containing perfume			x
Butylhydroxytoluene			x

* Diclofenac diethylamine emulsion gel 1.16% (Novartis, 2013a).

† Diclofenac diethylamine emulsion gel 2.32% (Novartis, 2013b).

### 2.2 Permeation and penetration into and across human skin

This *ex vivo* absorption method was based on the Organisation for Economic Cooperation and Development (OECD) Test No. 428: Skin absorption: *in vitro* method (OECD, 2004). Human abdominal skin samples from three patients were obtained via surgical skin removal procedures that were unrelated to the present investigation. Each of the three patients consented to the scientific use of skin prior to surgery. Skin was not used if a pathological finding was present or if there was skin damage, strongly marked scarring or pregnancy stretch marks.

The excised skin was cooled to 4 °C, the subcutaneous fatty layer was separated from the skin, and the skin specimen was stored at −20 °C until use. To prepare the skin sample for use, the specimen was thawed and cut into strips with a scalpel and dermatomized to a mean thickness of 500 ± 100 μm, leaving the stratum corneum intact. The suitability of skin from each donor was assessed by measuring the transdermal transport of caffeine to confirm that the skin samples remained relatively impermeable to this low permeability marker. This confirmation was performed using a Franz cell as described below. The caffeine solution (10 mg/ml) was added to the donor cell and 320 μl samples were withdrawn from the receiver compartment at 4, 6, 8, 20, 24, 28, 32, 46 and 48 hours. The sampled volume was replaced with phosphate‐buffered saline (PBS) after each sample was removed.

### 2.3 Franz cell

Circular samples from each skin specimen were prepared with a hollow punch and placed between the donor and receiver sections of a Franz cell, with the stratum corneum facing the donor cell. The receptor compartment had a volume of 12 ml and was filled with phosphate buffered saline pH 7.4 without Mg or Ca ions and was kept at 32 ± 1 °C. The receptor compartment was mixed with a magnetic stir bar at 400 rpm. About 200 mg of the test formulation was applied to the skin surface in the donor cell and the cell was sealed with Parafilm M (Brand, Germany) to prevent evaporation. Samples (320 μl) were taken from the receptor cell at 2, 5, 9, 24, 28, 32, 46 and 48 hours. The sampled volume was replaced with 320 μl PBS. Sink conditions, which are defined as a receptor concentration < 10% to 30% of the maximum solute concentration (European Medicines Agency, 2014), were maintained at all times based on a maximum observed diclofenac sodium concentration of 76 μg/ml, which was about 15% of the solubility of the two salts in 0.15 mM NaCl (about 520 μg/ml) (Khalil, et al., 2000).

At the conclusion of the permeation study, each skin sample was removed from the Franz cell and the residual drug delivery formulation was removed by gently swabbing with cotton swabs. The stratum corneum was removed by tape stripping as described by Wagner, et al., (2002). Each tape strip was placed in a vial for extraction of diclofenac or caffeine. Following tape stripping the skin sample was frozen at −80 °C in a cryomicrotome. Parallel 25 μm thick sections were cut and placed in a single vial for the extraction of diclofenac or caffeine.

### 2.4 Analytical methods

The extraction medium for diclofenac or caffeine was a 1:1 (v/v) mixture of ethanol and water. The tape strips were extracted in a minimum of 2 ml of medium and skin slices were extracted in 2 ml of medium. The extraction samples were shaken at 200 rpm for 60 min at room temperature. Diclofenac and caffeine concentrations were determined by HPLC methods that had been validated as described in the 2005 ICH Harmonised Tripartite Guideline ‘Validation of Analytical Procedures: Text and Methodology Q2(R1)’. The lower limit of quantification of diclofenac was 0.304 μg/ml. All assays were run in triplicate.

### 2.5 Calculations

The apparent permeation coefficient was calculated using Equation (1):

(1) $$P_{\mathrm{app}}=\frac{\mathrm{dQ}}{\mathrm{dt}}\;\cdot \;\frac{1}{m_{0}}\;\cdot \;\frac{1}{A}\;\cdot \;V_{D}\;$$

Where P_app_ is the apparent permeation coefficient (cm•s^−1^), dQ/dt is the steady state transport rate obtained by linear regression of the amount of test substance transported vs. time (μg/s), m_0_ is the initial mass of test substance in the donor compartment (μg), A is the area of exposed skin (cm^2^) and V_D_ is the donor volume (cm^3^).

### 2.6 Raman spectral imaging

Raman spectral imaging (Smith & Dent, 2005) was performed to assess the location and distribution of diclofenac in the three formulations; testing was performed by WITec (Wissenschaftliche Instrumente und Technologie GmbH, Ulm, Germany). Samples were prepared and placed between two coverslips and excited with a 532 nm diode laser. Single spectra were obtained separately from each component of each formulation. All Raman images and spectra were recorded using an alpha 300 RA Raman confocal imaging microscope (WITec) equipped with a CCD camera operating at −64 °C that captured an image 1600 pixels wide by 200 pixels high. The microscope was also equipped with an ultra‐high throughput spectrometer that had, in addition to the diode laser, 300 g/mm grating, BLZ = 500 nm, with a spectral centre of 2090 (rel 1/cm) and a Zeiss 100× oil immersion objective. Spectra were analysed with proprietary software (WITec ProjectFOUR and WITec ProjectFOUR Plus).

### 2.7 Statistical methods

Differences in cumulative transport into and through the skin samples were compared with a Mann–Whitney non‐parametric test. All results are expressed as mean ± standard deviation.

## 3 RESULTS

### 3.1 Ex vivo transport of diclofenac through human skin

Skin samples from three different female donors were used in these experiments. Samples were stored at −20 °C before use (220, 388 and 407 days, respectively). The P_app_ for caffeine was determined in four skin samples from each donor. The mean P_app_ for caffeine ranged from 3.04 × 10^−8^ cm•s^−1^ to 8.34 × 10^−8^ cm•s^−1^, values that were within the historically acceptable range for *ex vivo* permeation experiments in this laboratory (1.04 × 10^−8^ cm•s^−1^ to 9.38 × 10^−8^ cm•s^−1^) under the same conditions using the same methods.

**Figure 1:**
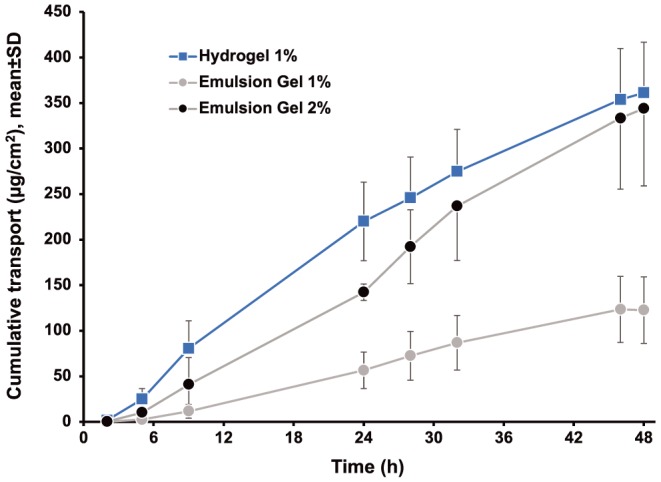
Transdermal transport of diclofenac through human skin ex vivo. Transdermal transport of diclofenac from three different formulations designed for topical administration. Cumulative transport corrected for skin surface area was calculated by measuring diclofenac concentration in the receptor medium at the time points indicated. Each point represents the arithmetic mean of nine determinations (three skin samples tested in triplicate). Statistical comparisons for the 5, 9 and 48 hour time points are shown in Figure 2

**Figure 2:**
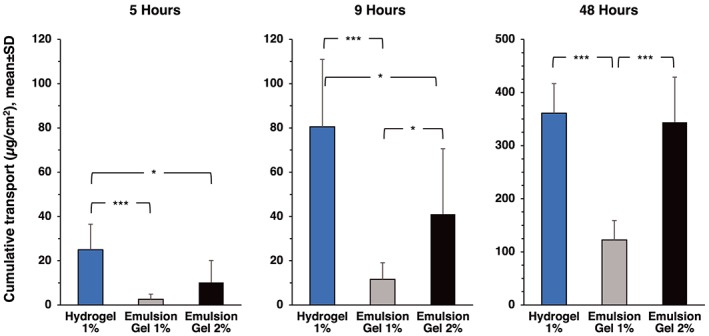
Analyses of cumulative transdermal transport of diclofenac from three formulations designed for topical administration. Each value represents the amount of diclofenac transported through the skin into the receptor medium. Statistically significant differences are shown by brackets (Mann–Whitney U test). *p < 0.05; ***p < 0.001

Figure 1 presents the cumulative diclofenac transdermal transport over time for each formulation and Figure 2 presents the analysis of cumulative transdermal transport of diclofenac from each formulation after 5, 9 and 48 hours. After 5 hours, the cumulative amount of diclofenac transported across skin from hydrogel 1% was about 10 times greater than from the emulsion gel 1% (*P*=0.0004) and more than twice the amount transported from emulsion gel 2% (*P*=0.022, Figures 1 and 2). Similar results were observed after 9 hours (Figures 1 and 2). The mean P_app_, which was derived from the slope of the cumulative transport curves, was 97.60 ± 9.83 × 10^−8^ cm•s^−1^ for hydrogel 1% compared with 34.93 ± 9.07 × 10^−8^ cm•s^−1^ and 47.10 ± 9.48 × 10^−8^ cm•s^−1^ for emulsion gel 1% and emulsion gel 2%, respectively (Table 2).

**Table 2:** The 48 hour mass balance of diclofenac after transport through human skin samples ex vivo

	Location	Emulsion gel 1%		Emulsion gel 2%		Hydrogel 1%	
		μg/cm^2^	%	μg/cm^2^	%	μg/cm^2^	%
Applied amount		1111.1 ± 0	100.0 ± 0	2222.2 ± 0	100.0 ± 0	1111.1 ± 0	100 ± 0
Not absorbed	Swab	771.1 ± 46.2	69.4 ± 4.2	1420.7 ± 66.0	63.9 ± 3.0	441.9 ± 56.6	39.8 ± 5.1
	First 2 tape strips	6.3 ± 2.7	0.6 ± 0.2	23.6 ± 11.9	1.1 ± 0.5	17.1 ± 13.2	1.5 ± 1.2
Absorbed	Stratum corneum	14.4 ± 3.7	1.3 ± 0.3	21.7 ± 2.6	1.0 ± 0.1	27.1 ± 11.9	2.4 ± 1.1
	Epidermis/dermis	3.8 ± 0.4	0.3 ± 0.04	12.4 ± 0.9	0.6 ± 0.04	7.3 ± 2.1	0.7 ± 0.2
	Receptor cell	122.6 ± 36.5	11.0 ± 3.3	343.9 ± 85.0	15.5 ± 3.8	360.9 ± 55.6	32.5 ± 5.0
Sum of absorbed and non‐absorbed		916.0 ± 21.9	82.4 ± 2.0	1818.1 ± 19.8	81.8 ± 0.9	849.4 ± 17.1	76.4 ± 1.5
Fraction absorbed		138.6 ± 35.9	12.5 ± 3.2	373.8 ± 89.9	16.8 ± 4.0	390.4 ± 52.5	35.1 ± 4.7
P_app_ (10^−8^ cm•s^−1^)		34.93 ± 9.07		47.10 ± 9.48		97.60 ± 9.83	

Values are presented as mean ± SD.

Table 2 presents a summary of the *ex vivo* transport of diclofenac after 48 hours. The calculated percent mass balance (the sum of absorbed and non‐absorbed diclofenac) was similar for each formulation and ranged from 76.4 ± 1.5% for hydrogel 1% to 82.4 ± 2.0% for emulsion gel 1%. The percentage of diclofenac absorbed from hydrogel 1% was 35.1 ± 4.7% compared with 12.5 ± 3.2% (*P*=0.0004) and 16.8 ± 4.0% (*P*=0.0004) from the emulsion gel 1% and emulsion gel 2%, respectively.

After 48 hours small amounts of diclofenac were found in the subsurface stratum corneum and the cryosections of skin for each formulation. The largest amount of diclofenac in the subsurface stratum corneum was found for hydrogel 1% (27.11 ± 11.87 μg/cm^2^), followed by emulsion gel 2% (21.70 ± 2.57 μg/cm^2^) and emulsion gel 1% (14.41 ± 3.70 μg/cm^2^). Smaller amounts of diclofenac were found in the dermal cryosections ranging from 3.79 μg/cm^2^ for emulsion gel 1% to 12.42 μg/cm^2^ for emulsion gel 2%.

### 3.2 Raman spectroscopy

**Figure 3:**
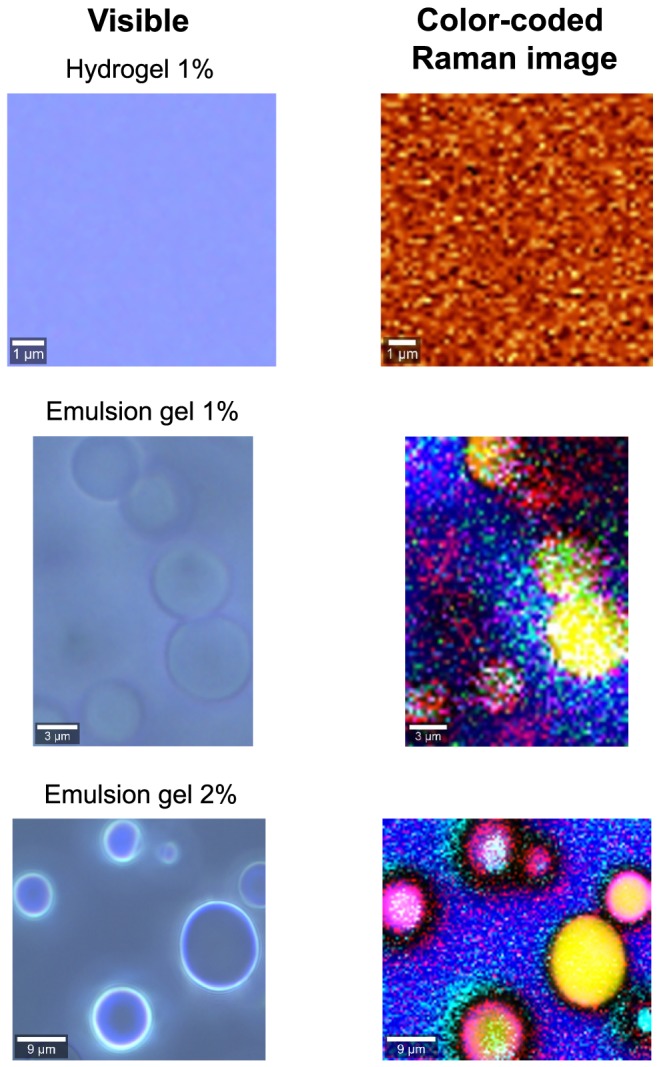
Raman spectroscopy. Visible images (left) and colour‐coded images (right) from Raman spectroscopy of the three diclofenac formulations. The Raman colour‐coded image of hydrogel 1% shows a homogeneous mixture of all formulation components. The emulsion gel 1% diclofenac, which is coded as red, appears primarily in association with paraffin, which is coded as yellow. A similar distribution of diclofenac is seen in the emulsion gel 2%

Colour‐coded Raman spectroscopy images of hydrogel 1%, emulsion gel 1% and emulsion gel 2% are presented in Figure 3. Important differences were seen in the hydrogel and emulsion gel formulations. The hydrogel 1% appeared as a homogeneous mixture of its various components, including diclofenac. In contrast, the Raman spectroscopy image of emulsion gel 1% revealed a non‐homogeneous mixture in which diclofenac was located in close proximity to the lipophilic phase (paraffin). The emulsion gel 2% sample also appeared to show diclofenac in proximity to paraffin.

## 4 DISCUSSION

The major finding of this *ex vivo* transdermal drug delivery study is that diclofenac from hydrogel 1% applied to human skin samples was more quickly transported across the skin than was diclofenac from emulsion gel 1% or 2%. This difference was clearly demonstrated by the mean P_app_ of diclofenac delivered from hydrogel 1%, which was 2.8‐fold greater and nearly 2.1‐fold greater than from emulsion gel 1% and emulsion gel 2%, respectively. Similar differences were also seen in the total amounts absorbed (Table 2). Importantly, this separation in efficiency of transdermal absorption was most evident after 5 hours, when 10 times more diclofenac had been transported across the skin from hydrogel 1% than from emulsion gel 1% (*P*=0.0004). The 5 hour time frame also revealed a substantial lag in the release and transdermal transport of diclofenac from the emulsion gel formulations. The faster onset and increased transdermal absorption rate of hydrogel 1% compared with the emulsion gel formulations suggest that hydrogel 1% would have a faster onset of action in underlying tissues compared with the emulsion gel products. The drug delivery advantage of hydrogel 1% was also evident at 9 hours, which is important because most topical NSAIDs are indicated to be applied 2 to 4 times daily (or about every 4 to 8 hours) (Novartis 2013a, 2013b).

The difference in transdermal delivery of diclofenac from hydrogel 1% and the two emulsion gel formulations observed in the *ex vivo* experiments described here is also likely to be seen during *in vivo* topical administration. In one of the original descriptions of this *ex vivo* method, Franz used his diffusion cell to compare the *ex vivo* human skin transit with *in vivo* transdermal absorption of a series of 12 randomly chosen organic compounds (Franz, 1975). Analysis of the total amount absorbed in the two tests revealed a significant rank‐order correlation (correlation coefficient = 0.734; p < 0.01). Further development of the method and harmonisation of its protocol has provided support for its use to establish bioequivalence between transdermal pharmaceutical formulations (Abd et al., 2016; Franz, et at., 2009). Based on the continuing evidence of good correlation between *in vivo* and *ex vivo* transdermal absorption rates and the agreement on bioequivalence of topical formulations using these test methods, regulatory agencies such as the European Medicines Agency and the United States Food and Drug Administration have encouraged their use as surrogates for *in vivo* bioequivalence studies, and a test method guidance has been published (OECD, 2004).

In order for diclofenac to be delivered to subcutaneous target tissues from a topically applied formulation it must release from the formulation into and diffuse through the lipophilic stratum corneum, through the underlying less lipophilic viable epidermis and ultimately through the dermis (Vitorino et al., 2015). The rate‐limiting step is the partitioning into the stratum corneum, which is in part influenced by the relative solubility of the drug in the formulation and in the stratum corneum (Vitorino et al., 2015). The overall rate of transdermal transport is a function of the diffusion coefficient of the drug in each compartment or layer as well as physical factors, including the viscosity and the length of the diffusion path (Vitorino et al., 2015). Importantly, the physical and chemical composition of the stratum corneum creates a barrier with anisotropic diffusion properties featuring lateral diffusion coefficients 40 to 300 times the transdermal diffusion coefficients (Nitsche, et al., 2019). In addition, diffusion through hair follicles may be an important transdermal pathway, especially for molecules with low lipid solubility (Barbero & Frasch, 2017).

In the present study, diclofenac more readily penetrated and transited the skin from the hydrogel formulation than from either of the emulsion formulations. One possible explanation for this difference was suggested by the Raman spectral analysis. In the hydrogel formulation diclofenac appears to exist in solution, whereas in the emulsion gel formulation diclofenac diethylamine resides as particles in the lipid phase of an oil‐in‐water emulsion within a polyacrylate‐based gel matrix. Thus diclofenac in the hydrogel is immediately available for diffusion into the skin compared with the emulsion gel in which diclofenac must first release from the lipid phase to be available for penetration of the skin (Seth, 1992). This concept is supported by work reported by Seth (1992), who compared the *in vivo* transdermal absorption of diclofenac sodium from a solution gel (an early developmental formulation of hydrogel 1%) and an emulsion gel (identified by the cited reference as diclofenac diethylamine 1.16%, Voltaren® Emulgel) in healthy volunteers. Diclofenac released from the solution gel demonstrated faster absorption as assessed by t_max_, 2‐fold higher peak plasma concentration and nearly a 2‐fold higher area under the curve (AUC) corrected for dose than diclofenac delivered from the emulsion gel. In addition, the water content of the hydrogel may increase hydration of the stratum corneum, which has been reported to cause morphological changes consistent with increasing the permeability of this barrier (Haftek et al., 1998; Marwah et al., 2016). Increased epidermal hydration is also reported to increase the permeability of the skin; for review see Marwah et al. (2016).

As illustrated in the present study, differences in the compositions of topical diclofenac formulations have been shown to be the primary determinants of differences in the rate of transdermal drug delivery (Brunner et al., 2011; Escribano et al., 2003; Folzer et al., 2014; Nivsarkar et al., 2015). For example, Escribano et al. (2003) compared the *ex vivo* human skin permeation of four distinct formulations of diclofenac sodium, including one microemulsion formulation. The transdermal flux of diclofenac from these formulations ranged from 5.7 μg/h to 147.7 μg/h, the microemulsion demonstrating the lowest flux and the three ternary solvent formulations exhibiting 3.8‐ to 26‐fold greater flux rates and corresponding differences in permeability coefficient and the amount of drug absorbed. The results of Escribano et al. suggest that certain combinations of solvents or permeation enhancers may act synergistically to enhance dermal permeation, specifically the combination of oleic acid and *D*‐limonene. Okuyama et al. reported that adding diisopropyl adipate, which is a component of the present hydrogel formulation, to a diclofenac alcohol gel synergistically enhanced the *in vivo* absorption of diclofenac in guinea‐pigs as shown by plasma AUC and by underlying muscle concentrations (Okuyama et al., 1999). Others have reported that formulating diclofenac as an acid rather than as a salt may enhance the transdermal absorption compared with diclofenac emulsion gel (Brunner et al., 2011). In addition, small differences in *ex vivo* transdermal permeation rates have been reported for different salts of diclofenac when tested in simple solutions (Minghetti, et al., 2007), but whether these differences occur in complex transdermal formulations has not been described.

Although oral NSAIDs remain a first‐line therapy for pain and inflammation of musculoskeletal disorders, including osteoarthritis, tolerability issues and safety concerns may limit their use in some patients (Henry & McGettigan, 2003; McGettigan & Henry, 2006, 2011). Topical application on the skin above the affected joint or tissue allows the delivery of an effective concentration of the drug locally while minimizing systemic exposure. For example, Brunner et al. (2005) measured diclofenac concentrations in plasma, subcutaneous adipose tissue and skeletal muscle following oral or topical administration in 12 healthy volunteers. After the final dose of a 3‐day treatment protocol, the concentrations of diclofenac were monitored in plasma, subcutaneous adipose tissue and skeletal muscle for the next 48 hours. The AUCs were 2.5 times higher in adipose tissue and 2.1 times higher in skeletal muscle than in plasma following topical drug administration compared with oral dosing. In contrast, the plasma AUC was 47.9 times higher following oral dosing than after topical administration. Müller et al. (1997) confirmed that topical administration of diclofenac resulted in drug concentrations exceeding 0.5 μg/ml (the cyclo‐oxygenase‐2 50% inhibitory concentration) in the underlying tissue layers in most treated subjects. Although the present study did not contain any *in vivo* pharmacokinetic measurements, it seems likely that a similar spectrum of exposures would be observed compared with oral dosing (i.e. sufficient local drug concentration combined with low systemic exposure compared with much higher systemic concentration following oral dosing).

The differences in *ex vivo* diclofenac permeation from the formulations in the present study may not translate directly to what might be observed in clinical use. The use of a Franz cell that is sealed with paraffin film limits solvent evaporation from the formulations during the test periods and thus does not mimic the intended clinical application in which the formulation remains unoccluded and subject to evaporation and erosion by clothing.

## 5 CONCLUSIONS

The results of this study indicate that hydrogel 1% was superior to emulsion gel 1% over 48 hours or emulsion gel 2%, particularly over the first 9 hours, in its ability to effectively deliver diclofenac into and through human skin *ex vivo*. The Raman spectral analysis suggests that differences in the partitioning of diclofenac within these formulations may in part explain the differences in the absorption rate. Although the skin absorption model is widely accepted for comparison of transdermal drug permeation, *in vivo* testing would be necessary to demonstrate that the *ex vivo* results translate to clinical use.

### ETHICS

Human abdominal skin samples from three patients were obtained via surgical skin removal procedures that were unrelated to the present investigation. Each of the three patients consented to the scientific use of skin prior to surgery. Tissue collection was carried out in compliance with the Declaration of Helsinki as currently amended. All participants provided informed consent at the time of surgical skin removal.

### PARALLEL USE OF COMPARATOR DATA

The emulsion gel comparator data used in this study also served as comparator data in a parallel study of a distinct diclofenac transdermal formulation. That work has now been published: Sacha, M., Faucon, L., Hamon, E., Ly, I. & Haltner‐Ukomadu, E. (2019). Ex vivo transdermal absorption of a liposome formulation of diclofenac. *Biomedicine and Pharmacotherapy, 111,* 785–790.

## DATA AVAILABILITY

Data are available upon request from the corresponding author.

## CONFLICT OF INTEREST

EHU, MS are employees of Across Barriers, which was contracted by ratiopharm to assist in this study. AR is an employee of WITec, which assisted in this study. KH was an employee of ratiopharm at the time of the study.
